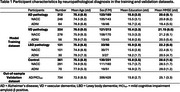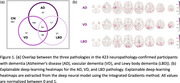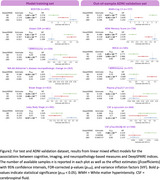# Detecting Pathology‐Confirmed Neurodegenerative Patterns in Mixed‐Dementia: Deep Learning Networks Construct Novel Neuroimaging Signatures of Alzheimer's, Vascular, and Lewy Body Dementias

**DOI:** 10.1002/alz70856_103182

**Published:** 2025-12-25

**Authors:** Di Wang

**Affiliations:** ^1^ UT Health Science Center at San Antonio, San Antonio, TX, USA

## Abstract

**Background:**

Clinical diagnosis of dementia faces accuracy challenges, with neuropathology as the diagnostic gold standard due to its ability to identify misfolded protein deposits with distinct spreading patterns. Deep learning has revolutionized biomarker development by achieving state‐of‐the‐art accuracy. However, most models were trained with clinical diagnoses, potentially limiting accuracy in predicting the correct dementia type. This study develops a multi‐label deep‐learning network trained on neuropathologically confirmed diagnoses to quantify atrophy across three common dementias: Alzheimer's, Vascular, and Lewy Body Dementias.

**Method:**

Models are trained using antemortem 3D T1‐weighted MRI scans from 423 demented participants and 361 controls from the NACC and ADNI datasets. The deep‐learning explainable heatmaps are generated to visualize pathology‐specific patterns and novel Deep Signature of Pathology Atrophy REcognition (DeepSPARE) indices are developed. A higher DeepSPARE score indicates greater brain alterations associated with a specific pathology. The DeepSPARE indices are tested with a variety of measures using linear mixed effect model. 734 out‐of‐sample ADNI validation subjects are used to validate the model performance

**Result:**

The deep‐learning model achieved the 5‐fold cross‐validated balanced accuracy of 0.844, 0.839, and 0.623 for AD, VD, and LBD, respectively. Explainable deep‐learning AD heatmap highlighted bilateral hippocampal regions, the VD heatmap emphasized white matter regions, and the LBD heatmap showed occipital alterations. The DeepSPARE indices were significantly associated with pathology‐specific measures. The DeepSPARE‐AD index was significantly associated with cognition, ADNC score, and Braak stages. The DeepSPARE‐VD index was significantly associated with white matter hyperintensity volume. The DeepSPARE‐LBD index was significantly associated with Lewy body stages. The out‐of‐sample ADNI dataset also confirmed such findings with cognition, imaging, CSF, and plasma measures.

**Conclusion:**

We have demonstrated the effectiveness of our novel deep‐learning framework in identifying antemortem neuroimaging signatures associated with distinct pathologies. The newly developed DeepSPARE indices are accurate, pathology‐specific, and noninvasive neuroimaging metrics, connecting widely available in‐vivo T1 imaging with pathological identifications.